# Strategies for remediating clinical reasoning skill deficits in underperforming residents: a scoping review

**DOI:** 10.3352/jeehp.2026.23.3

**Published:** 2026-02-05

**Authors:** Jovian Philip Swatan, Fithriyah Cholifatul Ummah, Cecilia Felicia Chandra, Nooreen Adnan

**Affiliations:** 1 Department of Neurology, Universitas Airlangga Hospital, Surabaya, Indonesia; 2 Department of Medical Education, Faculty of Medicine, Universitas Airlangga, Surabaya, Indonesia; 3 Medical Education, Research, and Staff Development Unit, Faculty of Medicine, Universitas Airlangga, Surabaya, Indonesia; 4 Dow Institute of Health Professionals Education, Dow University of Health Sciences, Karachi, Pakistan; 5 University of South Wales, Pontypridd, UK; Hallym University, Korea

**Keywords:** Clinical reasoning, Remedial teaching, Internship and residency, Scoping review

## Abstract

Clinical reasoning is a core competency in medical practice; however, deficits in this domain among residents are often difficult to identify and remediate because of its cognitive complexity and the absence of standardized assessment approaches. This scoping review aimed to map and analyze existing evidence on strategies to remediate clinical reasoning skill deficits in underperforming medical residents. Using the Arksey and O’Malley framework as refined by Levac and his colleagues, and reported in accordance with PRISMA-ScR (Preferred Reporting Items for Systematic Reviews and Meta-Analyses extension for Scoping Reviews) guidelines, we systematically searched PubMed, Scopus, MEDLINE, Web of Science, SpringerLink, ProQuest, and EBSCOhost for studies published between 2000 and 2024. Definitions of clinical reasoning, underperformance, and remediation were adopted from prior literature. Twenty studies met the inclusion criteria, comprising original research and literature reviews in multiple medical specialties. Methods for identifying clinical reasoning deficits included written, oral, and performance-based assessments, as well as routine workplace-based evaluations. Remediation strategies ranged from structured institutional programs to individualized, case-specific interventions, with coaching, deliberate practice, guided reflection, and structured thinking frameworks frequently employed. Two studies reported positive outcomes following completion of remediation for clinical reasoning deficits. Key enablers included psychological safety, learner engagement, and accessible faculty support, whereas barriers included learner resistance, inadequate baseline knowledge, faculty skill limitations, and institutional resource constraints. Effective remediation requires early identification, comprehensive diagnostic assessment, and tailored, coaching-based interventions supported by institutional commitment. Nonetheless, substantial variability in definitions, remediation protocols, and evaluation methods highlights the need for greater standardization and further research across diverse contexts to inform evidence-based frameworks for clinical reasoning remediation.

## Graphical abstract

[Fig f2-jeehp-23-03]

## Introduction

### Background

In today’s healthcare environment, physicians are confronted with an overwhelming volume of clinical data and increasingly complex decision-making demands [1]. In this context, strong clinical reasoning skills are more essential than ever to ensure the delivery of high-quality patient care. Clinical reasoning is a multifaceted process that integrates cognitive and interpersonal elements to support accurate diagnosis and effective management decisions, ultimately aimed at optimizing patient outcomes [2]. Central to this process is the physician’s ability to navigate uncertainty, which is an inherent and unavoidable feature of medical practice [3]. Physicians with a low tolerance for uncertainty may resort to excessive diagnostic testing or unnecessary treatments, thereby increasing healthcare costs and exposing patients to potential harm [3]. These observations underscore that while foundational knowledge is necessary, it is not sufficient on its own. Accordingly, fostering robust clinical reasoning abilities is a critical priority in medical education, particularly during the residency period.

Given that uncertainty is intrinsic to clinical practice, medical education must prepare learners not only to recognize uncertainty but also to manage it effectively [4]. However, traditional medical assessments have largely emphasized the recall of factual knowledge, pattern recognition, and algorithmic thinking, which tend to reflect certainty rather than the nuanced reasoning required in real-world clinical settings [5]. As a consequence, existing assessment methods may inadequately capture the depth and flexibility of a trainee’s clinical reasoning abilities. There is therefore an urgent need to shift educational practices toward fostering and assessing the capacity to reason through uncertainty and apply contextual judgment in clinical decision-making.

In this study, we used the clinical reasoning framework proposed by Daniel et al. [2] as the conceptual foundation to guide the review. This framework is grounded in a synthesis of several theoretical perspectives, including illness script theory, dual-process theory, and cognitive load theory, and incorporates insights from disciplines such as cognitive psychology, sociology, and education. Within this framework, clinical reasoning is defined as “a skill, process, or outcome wherein clinicians observe, collect, and interpret data to diagnose and treat patients.” This construct is further delineated into 7 domains: information gathering, hypothesis generation, problem representation, differential diagnosis, leading diagnosis, diagnostic justification, and management and treatment [2].

Errors in clinical reasoning may arise from 4 primary areas: inadequate knowledge, flawed data gathering, faulty data processing, or impaired metacognition [6]. For remediation to be effective, educators must be able to identify precisely where within this process a learner’s reasoning is breaking down, thereby enabling timely and targeted interventions. Early identification and intervention are critical to the development of competent, reflective, and safe practitioners [7]. Although a growing body of literature addresses strategies to improve clinical reasoning among medical students [5,6,8-10], comparatively little attention has been directed toward remediation approaches for underperforming residents.

At present, there is a paucity of evidence to inform best practices in the remediation of clinical reasoning, and no widely accepted framework exists to guide this process [11,12]. While some studies have reported success rates of up to 90% in addressing clinical reasoning skill deficits, skepticism persists among some clinical educators regarding the overall effectiveness and feasibility of remediation programs [12]. In addition, clinical reasoning difficulties are frequently conflated with deficits in medical knowledge rather than being recognized as a distinct competency domain, which further complicates their identification and remediation [11].

Given the limited literature on this topic and the considerable variability across educational systems and institutional contexts, a scoping review methodology was considered appropriate to map the existing evidence and synthesize available findings. To clarify the scope of this review, remediation was defined as “an intervention, or suite of interventions, required in response to assessment against threshold standards, with the aim of remedying underperformance so the doctor can return to safe practice” [13]. An underperforming resident was defined as “a trainee requiring additional intervention beyond the usual level of supervisor–trainee interaction in order to meet the expectations of their training level” [14].

### Objectives

This scoping review aims to map and analyze the existing evidence on strategies used to remediate clinical reasoning skill deficits in underperforming medical residents. Specifically, it seeks to address the following questions: (1) What tools or criteria are used to identify residents with clinical reasoning skill deficits? (2) What remediation strategies have been implemented to address clinical reasoning skill deficits in residents? (3) What are the outcomes of implemented remediation strategies for addressing clinical reasoning skill deficits? (4) What factors facilitate or hinder the successful remediation of clinical reasoning skills in residents?

## Methods

### Ethics statement

Ethical approval and written informed consent were not required because this scoping review involved only the analysis of previously published literature and did not include human participants or identifiable personal data.

### Study design

A scoping review methodology was conducted based on the framework outlined by Arksey and O’Malley [15] and further refined by Levac et al. [16] to examine the literature on remediation of clinical reasoning skill deficits in underperforming residents. The review followed 5 key stages: (1) identifying the research question; (2) identifying literature relevant to the research question; (3) selecting studies and extracting data; (4) analyzing the data; and (5) collating, summarizing, and reporting the findings. This scoping review was reported in accordance with the PRISMA-ScR (Preferred Reporting Items for Systematic Reviews and Meta-Analyses extension for Scoping Reviews) guidelines [17] (Supplement 1). A protocol for this scoping review was not registered.

### Eligibility criteria

The inclusion and exclusion criteria for this review were developed using the Population, Intervention, Comparison, Outcome, and Study Design (PICOS) framework, as detailed in Table 1. We included studies addressing clinical reasoning remediation programs within formal medical training or structured, assessed programs involving fully registered or licensed physicians across all specialties. Our exclusive focus on medical residents was based on the understanding that residency represents a critical period for identifying and remediating clinical reasoning deficits, thereby ensuring the development of robust skills necessary for navigating clinical uncertainty and providing safe, high-quality patient care as independent practitioners [4]. Studies involving mixed learner populations were eligible for inclusion if data specific to medical residents could be clearly identified and extracted. Review articles and meta-analyses reporting findings from primary studies were also included, whereas opinion papers and purely descriptive studies were excluded to ensure that the evidence synthesized in this review was grounded in empirical data and methodological rigor.

### Information sources

A comprehensive search was conducted in the electronic databases PubMed, Scopus, MEDLINE, Web of Science, SpringerLink, ProQuest, and EBSCOhost, with assistance from a librarian at the Faculty of Medicine, Universitas Airlangga. The search was performed in July 2025 and included studies published between January 2000 and December 2024.

### Search strategy

The search strategy for each database combined keywords and subject headings relevant to the PICOS framework. A sample search strategy is provided in Supplement 2. Filters were applied to include only full-text articles published in English between January 2000 and December 2024. The principal investigator (J.P.S.) and co-investigator (N.A.) supervised the consistent application of the search strategy across all databases. Articles were included if they addressed both remediation and clinical reasoning within the context of residency training.

After the initial database search, citation searching was conducted by the principal investigator (J.P.S.) through manual review of the reference lists of included studies. These reference lists were examined carefully to identify additional relevant articles that may not have been captured during the electronic searches. Any articles identified through this process were combined with those retrieved from the databases and subsequently screened using the same inclusion and exclusion criteria.

### Selection process

Following the search, all identified citations were imported into Mendeley Reference Manager for Windows ver. 2.135.0 (Elsevier Inc.), and duplicate records were removed. After full-text articles were retrieved with the assistance of a librarian, 2 reviewers (F.C.U. and C.F.C.) conducted an initial calibration exercise using the first 6 articles to ensure consistency in the screening process. After calibration, all titles, abstracts, and full-text articles were independently screened by the 2 reviewers. Discrepancies were resolved through discussion, with involvement of the principal investigator (J.P.S.) when consensus could not be reached.

### Data charting process

Following completion of the screening phase, the principal investigator and reviewers collaboratively identified key data elements to be extracted and designed an online data extraction form (Supplement 3). After consensus was reached on the data collection criteria, a second calibration exercise was conducted using the first 6 included articles to align reviewers’ approaches to data extraction. All included articles were independently extracted by 2 reviewers, and any discrepancies were resolved through discussion.

### Data items

Data extracted from eligible articles were descriptively summarized using frequency tables generated in Microsoft Excel (Microsoft Corp.). Extracted data included each article’s stated purpose or objectives, methods or tools used to identify clinical reasoning skill deficits, remediation strategies employed, reported outcomes, and barriers or enablers influencing implementation. The findings were analyzed using a narrative synthesis approach based on the framework described by Popay et al. [18] in 2006. This deductive approach involved establishing an initial conceptual framework to guide interpretation, developing a preliminary synthesis of findings, exploring patterns and relationships across studies, and assessing the robustness of the synthesis by considering study characteristics and methodological quality [18]. Mendeley Reference Manager (Elsevier) was used to organize, manage, and cite all referenced literature.

### Quality assurance

To ensure the quality of this scoping review, we adopted quality assurance procedures similar to those described in a prior remediation-focused review by Pirie et al. [19] in 2020. These procedures included calibration of reviewers during the initial screening, data abstraction, and coding phases. Calibration exercises were conducted to promote consistency and shared understanding of inclusion criteria, data extraction processes, and coding frameworks, and to clarify interpretation and categorization of extracted data.

Each article was screened independently by at least 2 reviewers. During the title and abstract screening stage, each reviewer independently assessed 6 articles using a classification system of “yes,” “no,” or “unsure” for inclusion. The principal investigator and reviewers then met to discuss decisions, resolve discrepancies, and address uncertainties. This process was repeated until a satisfactory level of agreement was achieved, after which reviewers proceeded with independent screening. The level of agreement between reviewers during initial screening was 85.7%.

A similar calibration and consensus process was applied during full-text review and data extraction, with an agreement rate of 90% between reviewers. Throughout the review process, articles that generated uncertainty were flagged for joint discussion. During the qualitative analysis phase, an additional calibration exercise was conducted to establish a shared coding framework, ensuring consistency in the identification of key themes and findings across included studies [19].

To further enhance rigor, the principal investigator assessed all included studies using the Modified Medical Education Research Study Quality Instrument (MMERSQI). MMERSQI was selected over the original Medical Education Research Study Quality Instrument (MERSQI) because it provides a more comprehensive assessment of study quality, including evaluation of risk of bias for randomized controlled trials, more detailed characterization of participant features, and a weighted scoring system that prioritizes performance-based assessments while accounting for the unequal contribution of individual quality domains [20].

The MMERSQI scoring protocol has been described previously. Briefly, a summary score is calculated by dividing the total score obtained by the total possible score, yielding a value between 0 and 1, with values closer to 1 indicating higher methodological rigor [20]. Although no established threshold exists to define minimum methodological adequacy, studies with higher MMERSQI scores were prioritized for outcome evaluation (Theme 3) due to their stronger methodological design.

## Results

### Study selection

A total of 2,046 records were identified through database searching. After removal of 464 duplicate records and 4 non-English records, 1,578 records remained for screening. Following title and abstract screening, 1,510 records were excluded. Full-text articles were sought for the remaining 68 records; however, one full-text article could not be retrieved, leaving 67 articles assessed for eligibility. Of these, 4 were excluded as descriptive or opinion papers, 16 did not include residents or physicians in training, 16 did not report remediation strategies, and 14 did not address clinical reasoning.

**Fig. 1.**
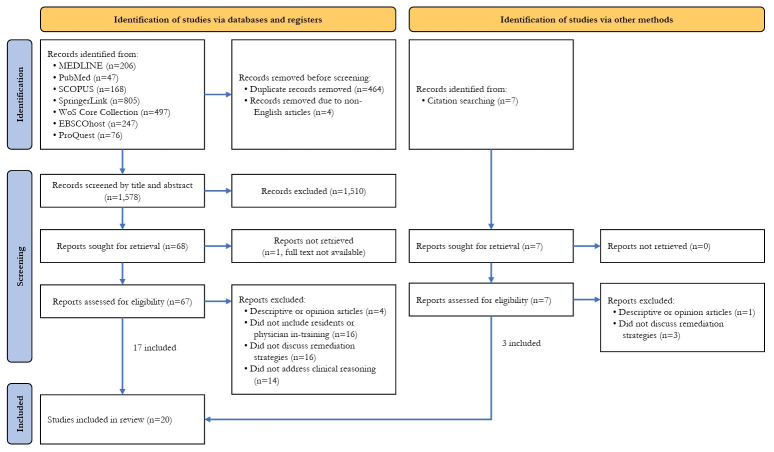
PRISMA (Preferred Reporting Items for Systematic Reviews and Meta-Analyses) flow diagram.

An additional 7 records were identified through citation searching. Full-text assessment of these records resulted in the exclusion of one descriptive or opinion paper and 3 articles that did not report remediation strategies. In total, 20 studies were included in the final review (Supplement 4). The study selection process is illustrated in the PRISMA flow diagram (Fig. 1).

### Study characteristics

Table 2 summarizes the characteristics of the 20 included studies [11,12,21-38]. Thirteen were original research articles, and 7 were literature reviews. Geographically, most studies originated from North America (n=14), with the remainder conducted in Europe (n=3), Australia (n=2), and Africa (n=1). Notably, the number of publications increased substantially after 2012, reflecting growing scholarly interest in this area.

Regarding clinical focus, 7 studies examined remediation programs spanning multiple disciplines, while others focused on internal medicine (n=4), emergency medicine (n=2), and one study each in family medicine, general surgery, neurosurgery, and otorhinolaryngology. Three studies did not specify a medical discipline. The included studies achieved a median MMERSQI score of 0.72 (interquartile range, 0.61–0.84; range, 0.24–0.91), indicating moderate overall methodological quality.

### Results of syntheses

#### Theme 1. Identifying residents with clinical reasoning skill deficits

Stakeholders involved in identifying clinical reasoning deficits: The stakeholders involved in identifying residents with clinical reasoning deficits are summarized in Table 3.

Methods for identifying clinical reasoning deficits: A variety of methods have been employed to identify residents with clinical reasoning deficits. These approaches can be broadly categorized into examination-based and non–examination-based methods. Examination-based methods were further divided into oral, written, and performance-based assessments. A summary of these methods is provided in Table 4.

#### Theme 2: Remediation of clinical reasoning skill deficits

Approaches to remediation: Remediation approaches ranged from structured, institutionally supported programs integrated into formal training frameworks [12,22,31,32,35] to unstructured, case-specific interventions implemented reactively and tailored to the specific clinical reasoning lapses identified [11,23,24,26,27,29,30,36,38].

The role of facilitators in the remediation of clinical reasoning skill deficits: Table 5 outlines the key stakeholders involved in the remediation of clinical reasoning skill deficits, while Table 6 summarizes the specific roles these facilitators assumed throughout the remediation process.

Remediation strategies to address clinical reasoning skill deficits: Several instructional strategies used to remediate deficits in clinical reasoning skills are summarized in Table 7. Overall, the selection and implementation of specific remediation strategies depended largely on institutional context, faculty expertise, and available resources. Notably, most reported remediation programs were conducted outside the formal residency curriculum.

Re-evaluation following remediation programs: Re-evaluation following completion of remediation was reported in 3 studies [12,31,35]. This process was typically conducted either by independent faculty members [12] or by the clinical competency committee responsible for overseeing the remediation program [31,35]. One study described the combined use of OSCEs, script concordance testing, mini clinical evaluation exercises (mini-CEX), and chart-stimulated recall as methods for post-remediation assessment [12].

#### Theme 3: Outcomes of clinical reasoning skill remediation

Nine studies reported outcomes of their remediation programs [12,22,30-35,38]. Across these studies, 55%–100% of residents successfully completed remediation and were able to finish their residency programs in good standing. In contrast, 4%–23% did not achieve good standing following remediation; these residents either transferred to another training program or exited residency altogether.

Only 2 studies specifically examined outcomes related to remediation of clinical reasoning skill deficits. Parsons et al. [31] in 2024 described a remediation program for residents and fellows with primary clinical reasoning deficits that incorporated clinical coaching, deliberate practice, formative feedback, and enhanced supervision. Of the 38 participants, 21 (55%) successfully graduated in good standing, 12 (32%) remained in their training programs with good standing, and 5 (13%) left the program without achieving good standing. In comparison, Guerrasio and Aagaard [12] in 2014 reported outcomes from a standardized remediation program emphasizing faculty guidance and coaching. Of the 53 participants, 51 (96%) successfully passed reassessment. Among these learners, 38 (72%) ultimately graduated or continued to practice in good standing. Six residents transferred to other residency programs, 4 were placed on probation and later graduated, 2 medical students were placed on probation and remain enrolled, and 1 resident pursued a non-clinical career path.

#### Theme 4: Enablers and barriers to the successful remediation of clinical reasoning skill deficits

Across the included literature, several factors influencing the success of remediation programs for clinical reasoning skill deficits were identified. These factors were broadly categorized into learner-related, tutor- or faculty-related, and institutional or policy-related domains. The enablers and barriers to successful remediation are summarized in Table 8 and Table 9, respectively.

## Discussion

Residency training represents a critical period for the early identification and targeted remediation of clinical reasoning deficits, as it is during this stage that learners consolidate clinical knowledge and transition toward independent practice. Despite the central role of remediation, much of the discourse surrounding competency-based medical education (CBME) has focused on high-achieving learners, with comparatively limited attention given to how CBME systems address learners who struggle or fail to meet competency expectations [39]. Research on remediation in medical education therefore remains underdeveloped. Over the past 3 decades, only approximately 100 studies on remediation in postgraduate medical programs have been published [40]. This scarcity likely reflects a combination of cultural, conceptual, methodological, and practical barriers, each contributing to the limited advancement of this important field.

### Identification of clinical reasoning deficits

Educators and clinical supervisors were most frequently responsible for identifying lapses in clinical reasoning among residents. This is appropriate given their close involvement in daily supervision, including case discussions, which allows them to recognize subtle cues suggestive of reasoning difficulties [28]. However, several studies have reported instances in which poorly performing residents were not identified by supervisors, often due to limited awareness of performance cues or inadequate documentation of concerns [41]. This gap may reflect insufficient training in recognizing clinical reasoning deficits, as well as the inherent subjectivity and variability of supervisory judgments, which are frequently shaped by individual experience and applied inconsistently across learners [28].

Although some studies reported resident self-referral for remediation, these cases typically accounted for fewer than 10% of referrals [31-33]. Residents who self-referred demonstrated a comparable number of clinical reasoning deficiencies to those identified by faculty or program directors [32], suggesting that such learners often possess greater insight into their own performance. Nonetheless, reliance on self-referral alone is insufficient. Underperforming learners may inaccurately assess their abilities, frequently overestimating their performance, which can delay identification and remediation of deficits [42]. Furthermore, the culture of perfectionism and sustained high achievement that characterizes many residency programs may foster an environment in which deviations from expected standards are perceived as personal failures [43]. This cultural context can discourage residents from acknowledging difficulties and seeking support [33], particularly among those experiencing psychological distress or burnout [44]. In light of these challenges, institutions should adopt proactive mechanisms to identify and support struggling learners rather than relying solely on self-referral.

Drawing on the clinical reasoning framework proposed by Daniel et al. [2], assessment of clinical reasoning can be aligned with specific reasoning domains. For example, Daniel et al. [2] highlight that think-aloud methods permit assessment across multiple clinical reasoning domains. This observation is consistent with findings by Parsons et al. [31], who reported that think-aloud exercises and case-based examinations are effective in identifying deficits in specific clinical reasoning microskills, including hypothesis generation, data gathering, problem representation, hypothesis refinement, and management planning. Beyond informing structured remediation programs, this microskill-based approach may also support real-time assessment and teaching by clinical educators, enabling targeted feedback, early recognition of reasoning gaps, and timely instructional adjustments during routine clinical encounters [31].

Accordingly, no single assessment method is sufficient to comprehensively evaluate all domains of clinical reasoning. Exclusive reliance on high-stakes examinations has important limitations. Although such examinations are valued for their structured format and reduced subjectivity [45], their reliability may still be influenced by factors such as performance anxiety, unfamiliarity with examination formats, fatigue, and other contextual variables that may not accurately reflect a resident’s underlying reasoning ability [46]. Conversely, assessment of clinical reasoning through daily workplace performance may reveal higher rates of error or critical incidents, including inaccurate problem identification, misdiagnosis, or inappropriate management decisions. However, there is no standardized threshold to determine whether such events reflect genuine reasoning deficits or are attributable to transient stressors or unrelated performance issues [47]. Integrating examination-based and non-examination-based assessments is therefore essential to developing a more comprehensive and accurate understanding of struggling learners. A combined approach facilitates earlier identification of deficits and supports timely, targeted intervention. In this context, using both types of assessments represents the most effective strategy for identifying weaknesses in clinical reasoning skills.

### Remediation of clinical reasoning skill deficits

A central issue in the remediation of clinical reasoning deficits is whether they should be addressed as a distinct focus or managed concurrently with deficiencies in other competency domains. Clinical reasoning deficits frequently coexist with gaps in medical knowledge or other performance concerns, which can exacerbate overall difficulties if not addressed systematically [12,28]. For this reason, remediation should begin with a comprehensive diagnostic assessment to identify the specific constellation of issues affecting each resident. When appropriate, addressing clinical reasoning as a discrete focus enables educators to apply targeted interventions that directly address the underlying cognitive processes involved in reasoning, rather than diffusing efforts across multiple domains simultaneously [28]. Such an approach also promotes efficient use of limited educational resources and maximizes the potential impact of remediation strategies [11,31]. Notably, clinical reasoning deficits often require longer remediation periods than other competency domains, reflecting the complex and integrative nature of this skill [31,32,35]. This further underscores the need for deliberate, individualized, and strategically sequenced remediation strategies rather than generic or parallel interventions by default.

Both Warburton et al. [35] in 2017 and Guerrasio and Aagaard [12] in 2014 describe comprehensive remediation frameworks that emphasize systematic and individualized approaches. Given the complexity and resource-intensive nature of remediation, coaching plays a central role in addressing clinical reasoning deficits [12,28,30,31,35]. In contrast to traditional didactic instruction, coaching in medical education is characterized by a collaborative partnership that prioritizes individualized learning needs and professional development [48]. Case-based coaching allows learners to engage with authentic clinical scenarios while practicing hypothesis generation, data gathering, differential diagnosis, and management planning under guided supervision [24,26,29,31,38]. Deliberate practice further reinforces these skills by providing repeated, targeted exercises accompanied by timely feedback to refine reasoning strategies [11,26,29,31,35,37]. Additional approaches include the use of structured reasoning frameworks or diagnostic algorithms to support decision-making [11,26,29,32,38], guided reflection to consolidate learning [11,12,26,29,35], and verbalization of thought processes to make reasoning explicit and amenable to feedback [11,28,32].

Overall, implementation of coaching-based or faculty-guided remediation for clinical reasoning deficits has been associated with positive outcomes [12,31]. Residents frequently report favorable perceptions of facilitators acting as coaches, highlighting accessibility, approachability, increased productivity, and honest feedback as key factors supporting their learning and progression [32]. However, establishing and sustaining such initiatives requires substantial institutional investment, including protected faculty time, sustained engagement, and financial resources [32]. These demands may limit feasibility in programs with constrained staffing or funding, particularly in the absence of dedicated remediation committees, and may increase the workload of already overextended faculty members [31].

Despite the growing use of targeted remediation approaches, mapping specific remediation strategies to the clinical reasoning domains proposed by Daniel et al. [2] remains challenging. This difficulty stems from substantial variation in remediation practices and implementation across educational centers, as well as from the fact that several studies did not explicitly state the clinical reasoning framework underpinning their remediation interventions. Consequently, alignment between remediation strategies and individual clinical reasoning domains is often implicit rather than explicit, which limits comparability across programs and studies.

Re-evaluation of clinical reasoning skills following completion of remediation programs is essential to determine whether interventions have achieved their intended goals [12,31]. Ideally, reassessment should be conducted by independent faculty members who are unaware that the learner has undergone remediation, as this approach enhances objectivity and supports fair judgment regarding attainment of expected competency standards [12]. In contrast, reassessment conducted by faculty who delivered the remediation may introduce valuation bias [49]. Although these faculty members can provide valuable insight into learner progress, their judgments may be influenced by perceived improvement rather than verified competence [35]. Passing post-remediation evaluation is strongly associated with successful completion of residency training [12]. Conversely, failure to meet competency standards after remediation places additional burdens on both learners and faculty, including heightened stress for learners and increased workload and resource demands for educators [41]. Taken together, post-remediation re-evaluation represents a critical step in verifying readiness for continued training and safe independent practice.

### Enablers and barriers to successful remediation of clinical reasoning skill deficits

The success of remediation is influenced by a combination of internal (learner-related) and external (faculty- or tutor-related and institutional or policy-related) factors. Among these, learner-related factors are most frequently identified as significant barriers to effective remediation of clinical reasoning skill deficits [28-30,37]. This is not unexpected, as the learner is the primary agent of change, and remediation programs aim to support affective, cognitive, and metacognitive development [50]. Challenges such as insufficient baseline clinical knowledge, psychological distress, burnout, and resistance to feedback can substantially limit the effectiveness of remediation efforts [28-30,37].

Establishing a transparent and open relationship between faculty and learners is a key enabler of successful remediation [23,32]. This can be achieved by actively involving learners in the development of remediation plans, reaching a shared understanding of goals and expectations prior to program initiation [12], providing clear and constructive feedback, and fostering a supportive and approachable learning environment [31]. Such collaboration ensures that remediation efforts address both clinical reasoning deficits and broader learner well-being. Additional supportive measures include temporary reduction of clinical responsibilities during remediation [24,32] and clear separation of coaching roles from formal assessment duties. Together, these strategies help create a psychologically safe learning environment that prioritizes growth and development rather than punitive oversight [31].

Faculty- and tutor-related factors represent another major barrier to effective remediation. These include limited expertise in identifying and addressing clinical reasoning deficits [11,27,38], as well as constrained capacity or willingness to engage in remediation activities [11]. In some cases, supervisors may oversimplify performance problems by attributing them solely to organizational issues or knowledge gaps, without undertaking a more nuanced diagnostic assessment [12,28]. This can perpetuate a cycle in which underperforming residents are not promptly identified or effectively remediated, increasing the risk of persistent deficits and potential patient safety concerns. Addressing these challenges requires institutional and policy-level support, including implementation of structured or standardized remediation frameworks [11,35,38], appointment of dedicated remediation coordinators [38], and provision of targeted faculty development programs to build remediation expertise [28].

### Strengths and limitations

To the best of the authors’ knowledge, this scoping review is the first to specifically examine remediation of clinical reasoning skill deficits among underperforming medical residents. The review offers a comprehensive synthesis of existing literature, encompassing identification methods, remediation strategies, reported outcomes, and factors influencing remediation success. The use of a systematic search strategy and a rigorous quality assurance process for included studies further strengthens the credibility and transparency of the findings.

Nevertheless, several limitations warrant consideration. First, the narrow scope of this review resulted in the inclusion of only 20 studies. Given the limited evidence base and qualitative nature of the review, all eligible studies were retained despite variability in methodological quality, including some with low MMERSQI scores. Second, most included studies originated from North America and Europe, with no representation from Asia. As approaches to identifying underperformance and implementing remediation may vary across sociocultural and institutional contexts, caution is needed when applying these findings to other settings. Finally, the lack of consensus regarding definitions of clinical reasoning deficits, remediation design, and outcome evaluation complicates alignment with specific reasoning domains and limits comparison of remediation effectiveness across studies.

### Future directions

Further research is needed to strengthen the evidence base for remediation of clinical reasoning skill deficits in underperforming residents. First, the development of a standardized definition of clinical reasoning, along with a clear articulation of its multidimensional domains, would promote conceptual clarity and consistency in reporting. Second, creation and testing of remediation frameworks grounded in shared definitions, supported by pilot studies evaluating specific strategies, would provide more robust evidence to guide best practices. Finally, increased contributions from underrepresented regions would enhance the generalizability of findings and support adaptation of remediation approaches across diverse educational and cultural contexts.

## Conclusion

This scoping review maps a limited but evolving body of literature addressing remediation of clinical reasoning skill deficits among underperforming medical residents. The included studies commonly describe approaches involving early identification of difficulties, individualized assessment, and structured educational interventions, most often incorporating coaching and elements of deliberate practice. Facilitators are frequently positioned in coaching roles that support reflective learning and progressive development of clinical reasoning processes. Institutional factors—such as faculty engagement, structured remediation systems, and attention to learner support—are also consistently highlighted as influencing implementation.

However, substantial variability in definitions, conceptual frameworks, methodological approaches, and outcome measures limits direct comparison across studies and constrains conclusions regarding effectiveness. Many reports describe remediation practices without explicit alignment to established clinical reasoning frameworks. From a practical standpoint, educators and training programs may consider adopting existing clinical reasoning frameworks to structure assessment and remediation while remaining responsive to local context and resource constraints. Further empirical research is required to promote consistency, comparability, and evidence-based advancement in this area.

## Figures and Tables

**Figure f2-jeehp-23-03:**
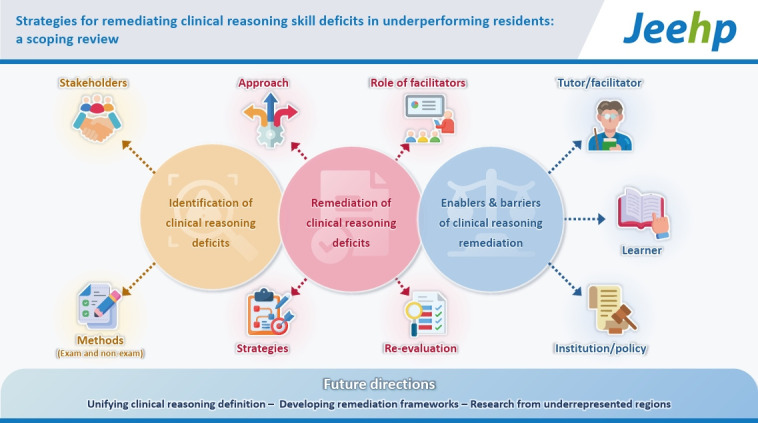


**Table 1.** PICOS framework for this study

PICOS categories	Inclusion criteria	Exclusion criteria
Population	Residents or physicians in training—defined as fully licensed physicians who have completed pre-registration requirements and are currently enrolled in formal training programs.	• Specialists who have completed their training.
		• Physicians who have completed or withdrawn from training programs.
		• Physicians not enrolled in a formal training program.
		• Undergraduate medical students.
		• Allied health professionals (e.g., pharmacists, dietitians, chiropractors, midwives, podiatrists, speech-language therapists, occupational therapists, physiotherapists).
		• Individuals from non-clinical or non-medical disciplines (e.g., clinical and translational research).
		• Practitioners of complementary, traditional, veterinary, or dental medicine.
Intervention	Remediation programs conducted in academic, clinical, or professional settings within a training program that specifically address clinical reasoning skills.	• Remediation processes that are poorly described or lack sufficient detail.
		• Interventions that do not explicitly target clinical reasoning skills.
Comparison	Comparison of various practices in remediation programs, including remediation approaches, modalities, objectives, processes, and the presence of enabling factors or barriers.	
Outcome	The main outcome of interest is the effectiveness of remediation programs in improving clinical reasoning skills among residents.	
Study design	• Articles published in English or translated into English.	• Descriptive papers, opinion pieces, and grey literature.
	• Study designs including: mixed methods research, meta-analyses, systematic reviews, review articles, randomized controlled trials, cohort studies, case-control studies, and cross-sectional studies.	
	• Publication years: 2000–2024	
	• Databases: PubMed, Scopus, Medline, Web of Science, SpringerLink, ProQuest and EBSCOhost.	

**Table 2.** Summary of included studies

Authors (year)	Country	Study design	Population	Study focus^a)^				MMERSQI
				1	2	3	4	
Dickinson et al. [21] (2009)	Australia	Literature review (consensus paper)	NA	✔	✔			0.72
Roy et al. [22] (2016)	USA	Literature review	NA	✔	✔		✔	0.68
Audetat et al. [11] (2017)	Canada, Switzerland	Literature review (consensus paper)	NA	✔	✔		✔	0.69
Nadir et al. [23] (2019)	USA	Literature review (consensus paper)	NA	✔	✔		✔	0.68
Jensen et al. [24] (2021)	USA	Literature review	NA	✔	✔		✔	0.24
Ekpenyong et al. [25] (2024)	USA	Literature review	NA	✔	✔		✔	0.84
Yan et al. [26] (2022)	USA	Meta-analysis	NA	✔	✔			0.91
Bond et al. [27] (2008)	USA	Qualitative focus groups	Consensus group from the 2008 Academic Emergency Medicine Consensus Conference	✔	✔		✔	0.56
Audetat et al. [28] (2012)	Canada, Belgium, Switzerland	Qualitative focus groups	Four focus groups with 26 clinical educators in general practice, internal medicine, and emergency medicine (multidisciplinary)	✔	✔		✔	0.81
Melia et al. [29] (2020)	USA	Qualitative focus groups	Members of the Infectious Diseases Society of America Training Program Directors’ Committee (internal medicine)	✔	✔		✔	0.56
Aram et al. [30] (2013)	Australia	Quantitative, retrospective cross-sectional	189 interns who took part in clinical rotations at Royal Brisbane and Women’s Hospital (multidisciplinary)	✔	✔	✔	✔	0.84
Parsons et al. [31] (2024)	USA	Quantitative, retrospective cross-sectional	114 residents and fellows with performance concerns; 38 with deficiency in clinical reasoning (multidisciplinary)	✔	✔	✔	✔	0.62
Guerrasio et al. [32] (2014)	USA	Quantitative, prospective cohort	151 learners (including medical students, residents, fellows, and attending physicians) either self-referred or were referred to the remediation program director (multidisciplinary)	✔	✔	✔	✔	0.89
Guerrasio and Aagard [12] (2014)	USA	Quantitative, prospective cohort	151 learners (including medical students, residents, fellows, and attending physicians) either self-referred or were referred to the remediation program director; 53 with clinical reasoning deficits (multidisciplinary)	✔	✔	✔	✔	0.89
Yao and Wright [33] (2000)	USA	Quantitative, prospective cross-sectional	404 internal medicine program directors	✔	✔		✔	0.83
Bhatti et al. [34] (2016)	USA	Quantitative, prospective cross-sectional	106 otorhinolaryngology program directors	✔	✔	✔	✔	0.59
Warburton et al. [35] (2017)	USA	Quantitative, prospective cross-sectional	14 graduate medical education learners referred to the early intervention remediation committee (multidisciplinary)	✔	✔	✔	✔	0.73
Naude and Burch [36] (2018)	South Africa	Quantitative, prospective cross-sectional	88 medical residents and 30 clinician-educators involved in the examination (internal medicine)	✔	✔		✔	0.81
Boyle et al. [37] (2024)	USA	Quantitative, prospective cross-sectional, instrument validation and testing	76 nephrology fellows (internal medicine)	✔	✔		✔	0.59
Audetat et al. [38] (2015)	Canada, Switzerland	Mixed-methods	21 family medicine residents in academic difficulty		✔	✔	✔	0.73

MMERSQI, Modified Medical Education Research Study Quality Instrument; NA, not applicable.

a) Study focus: (1) identification of residents with clinical reasoning skill deficits; (2) remediation of clinical reasoning deficits; (3) outcomes of clinical reasoning skill remediation; and (4) enablers and barriers of successful remediation of clinical reasoning skill deficits.

**Table 3.** Stakeholders involved in identifying residents with clinical reasoning deficits

Stakeholders	References
Allied health professionals	[34]
Educators and assessors (e.g., faculty, supervisors, examiners)	[11,12,22-24,26-37]
Peers	[33,34]
Program directors	[12,23,29,31-34]
Remediation assessment committee	[25,26,35]
Self-identified or self-referred	[12,22,31,32]

**Table 4.** Methods for identifying residents with clinical reasoning deficits

Methods	References
Examination-based	
Oral	
Case-based assessments	[11,24,29,31,33]
Think-aloud exercises	[27,31]
Written	
Script concordance tests	[12,21]
Written simulated case evaluations	[37]
Performance-based	
Objective structured clinical examinations	[34]
Direct observations	[11,12,27,28,33,35,36]
Simulation exercises	[21,23,27]
Non–examination-based	
Interviews to explore reasoning gaps	[12,22,26,32,35]
Chart reviews	[12,26,30,32,35,37]
Performance evaluations during ward rounds	[24]
Retrospective video reviews of clinical interactions	[21]
Intuitive recognition of poor performance cues	[28,33]
Monitoring of critical incidents	[33]
Multisource feedback	[21]

**Table 5.** Stakeholders involved in the remediation of clinical reasoning skill deficits

Stakeholders	References
Program directors	[23,37]
Remediation specialist or committee	[22,25,26,31,32,35]
Supervisors and faculty members	[11,12,22-32,36,38]

**Table 6.** Roles assumed by facilitators in the remediation of clinical reasoning skill deficits

Facilitator role	References
Guide or coach	[11,12,22,26,29-32,35-38]
Role model	[11,24,28]
Supervisor or assessor	[23,24,27,33,35,38]

**Table 7.** Remediation strategies to address clinical reasoning skill deficits

Remediation strategies	References
Pedagogical approach	
Case-based coaching or case reviews	[24,26,29,31,38]
Deliberate practice exercises	[11,26,29,31,37]
Guided reflective practice	[11,12,26,29]
Think-aloud or verbalization of reasoning processes	[11,28,32]
Structured reasoning frameworks or diagnostic algorithms	[11,26,29,32,38]
Delivery modalities	
Simulation-based training or exercises	[23,26,38]
Supplementary reading and learning resources	[24,29]
Video-assisted clinical reasoning reviews	[11,26]
Written case analysis or management worksheets	[26]

**Table 8.** Enablers to the successful remediation of clinical reasoning skill deficits

Enablers	References
Tutor- or faculty-related factors	
Identification of specific clinical reasoning microskills	[11,29,31,34]
Use of effective feedback techniques	[29]
Institutional- or policy-related factors	
Separation of the coaching role from formal assessment responsibilities	[31]
Implementation of structured or standardized remediation programs	[11,35,38]
Reduction of clinical responsibilities during remediation	[24,32]
Transparency regarding remediation program details provided in advance	[23,32]
Early enrolment in remediation programs	[29,32]
Assessment and reassessment conducted by faculty members not directly involved in remediation	[24]
Appointment of a dedicated remediation coordinator	[38]
Collaboration among key stakeholders	[23]
Adequate training for faculty involved in remediation programs	[28]

**Table 9.** Barriers to successful remediation of clinical reasoning skill deficits

Barriers	References
Learner-related factors	
Poor baseline clinical knowledge	[37]
High clinical workload	[28,29]
Reluctance to seek help and resistance to feedback	[30]
Tutor- or faculty-related factors	
Limited capacity or willingness of teaching staff	[11]
Difficulty distinguishing between minor errors and deficits requiring remediation	[11,27]
Lack of a strong theoretical foundation for remediation strategies	[38]
Inappropriate use of diagnostic or intervention tools for reasoning deficits	[30,38]
High clinical workload	[28,29]
Institutional- or policy-related factors	
Time constraints and prolonged remediation periods	[12,22,23,25,27,29,30,32,34-37]
Absence of structured or standardized remediation programs	[28]
Concerns among program directors regarding potential legal implications	[35]
Inadequate training of clinical educators in remediation	[28]

## Supplementary materials

Supplement 1. Preferred Reporting Items for Systematic Reviews and Meta-Analyses extension for Scoping Reviews (PRISMA-ScR) Checklist.

Supplement 2. Detailed search strategy.

Supplement 3. Data extraction form.

Supplement 4. Summary table of included studies.

Supplement 5. Audio recording of the abstract.

## References

[1] Higgs J, Grace S, Higgs J, Jensen GM, Loftus S, Trede FV, Grace S (2025). Clinical reasoning in the health professions.

[2] Daniel M, Rencic J, Durning SJ, Holmboe E, Santen SA, Lang V, Ratcliffe T, Gordon D, Heist B, Lubarsky S, Estrada CA, Ballard T, Artino AR, Sergio Da Silva A, Cleary T, Stojan J, Gruppen LD (2019). Clinical reasoning assessment methods: a scoping review and practical guidance. Acad Med.

[3] Brun C, Zerhouni O, Akinyemi A, Houtin L, Monvoisin R, Pinsault N (2023). Impact of uncertainty intolerance on clinical reasoning: a scoping review of the 21st-century literature. J Eval Clin Pract.

[4] Cooke S, Lemay JF (2017). Transforming medical assessment: integrating uncertainty into the evaluation of clinical reasoning in medical education. Acad Med.

[5] Connor DM, Durning SJ, Rencic JJ (2020). Clinical reasoning as a core competency. Acad Med.

[6] Cutrer WB, Sullivan WM, Fleming AE (2013). Educational strategies for improving clinical reasoning. Curr Probl Pediatr Adolesc Health Care.

[7] Rumack CM, Guerrasio J, Christensen A, Aagaard EM (2017). Academic remediation: why early identification and intervention matters. Acad Radiol.

[8] Brentnall J, Thackray D, Judd B (2022). Evaluating the clinical reasoning of student health professionals in placement and simulation settings: a systematic review. Int J Environ Res Public Health.

[9] Schmidt HG, Mamede S (2015). How to improve the teaching of clinical reasoning: a narrative review and a proposal. Med Educ.

[10] Linn A, Khaw C, Kildea H, Tonkin A (2012). Clinical reasoning: a guide to improving teaching and practice. Aust Fam Physician.

[11] Audétat MC, Laurin S, Dory V, Charlin B, Nendaz MR (2017). Diagnosis and management of clinical reasoning difficulties: Part II. Clinical reasoning difficulties: management and remediation strategies. Med Teach.

[12] Guerrasio J, Aagaard EM (2014). Methods and outcomes for the remediation of clinical reasoning. J Gen Intern Med.

[13] Price T, Wong G, Withers L, Wanner A, Cleland J, Gale T, Prescott-Clements L, Archer J, Bryce M, Brennan N (2021). Optimising the delivery of remediation programmes for doctors: a realist review. Med Educ.

[14] Barrett A, Galvin R, Steinert Y, Scherpbier A, O'Shaughnessy A, Horgan M, Horsley T (2015). A BEME (Best Evidence in Medical Education) systematic review of the use of workplace-based assessment in identifying and remediating poor performance among postgraduate medical trainees. Syst Rev.

[15] Arksey H, O’Malley L (2005). Scoping studies: towards a methodological framework. Int J Soc Res Methodol.

[16] Levac D, Colquhoun H, O'Brien KK (2010). Scoping studies: advancing the methodology. Implement Sci.

[17] Tricco AC, Lillie E, Zarin W, O'Brien KK, Colquhoun H, Levac D, Moher D, Peters MDJ, Horsley T, Weeks L, Hempel S, Akl EA, Chang C, McGowan J, Stewart L, Hartling L, Aldcroft A, Wilson MG, Garritty C, Lewin S, Godfrey CM, Macdonald MT, Langlois EV, Soares-Weiser K, Moriarty J, Clifford T, Tunçalp Ö, Straus SE (2018). PRISMA Extension for Scoping Reviews (PRISMA-ScR): checklist and explanation. Ann Intern Med.

[18] Popay J, Roberts H, Sowden A, Petticrew M, Arai L, Rodgers N, Britten A, Roen K, Duffy S (2006). Guidance on the conduct of narrative synthesis in systematic reviews: a product from the ESRC Methods Programme.

[19] Pirie J, St Amant L, Glover Takahashi S (2020). Managing residents in difficulty within CBME residency educational systems: a scoping review. BMC Med Educ.

[20] Al Asmri M, Haque MS, Parle J (2023). A Modified Medical Education Research Study Quality Instrument (MMERSQI) developed by Delphi consensus. BMC Med Educ.

[21] Dickinson I, Watters D, Graham I, Montgomery P, Collins J (2009). Guide to the assessment of competence and performance in practising surgeons. ANZ J Surg.

[22] Roy B, Chheda SG, Bates C, Dunn K, Karani R, Willett LL (2016). For the general internist: a summary of key innovations in medical education. J Gen Intern Med.

[23] Nadir NA, Hart D, Cassara M, Noelker J, Moadel T, Kulkarni M, Sampson CS, Bentley S, Naik NK, Hernandez J, Krzyzaniak SM, Lai S, Podolej G, Strother C (2019). Simulation-based remediation in emergency medicine residency training: a consensus study. West J Emerg Med.

[24] Jensen RL, Kestle JRW, Brockmeyer DL, Couldwell WT (2021). Principles of remediation for the struggling neurosurgery resident. World Neurosurg.

[25] Ekpenyong A, Holmboe ES, Govaerts M, Heeneman S (2024). Investigating the roles and impact of clinical competency committees in graduate medical education: a narrative review. J Grad Med Educ.

[26] Yan Q, Treffalls RN, Li T, Prasla S, Davies MG (2022). Graduate Medical Education “Trainee in difficulty” current remediation practices and outcomes. Am J Surg.

[27] Bond W, Kuhn G, Binstadt E, Quirk M, Wu T, Tews M, Dev P, Ericsson KA (2008). The use of simulation in the development of individual cognitive expertise in emergency medicine. Acad Emerg Med.

[28] Audétat MC, Dory V, Nendaz M, Vanpee D, Pestiaux D, Junod Perron N, Charlin B (2012). What is so difficult about managing clinical reasoning difficulties?. Med Educ.

[29] Melia MT, Paez A, Reid G, Chirch LM, Luther VP, Blackburn BG, Perez F, Abdoler E, Kaul DR, Rehm S, Harik N, Barsoumian A, Person AK, Yun H, Beckham JD, Boruchoff S, Cariello PF, Cutrell JB, Graber CJ, Lee DH, Maziarz E, Paras ML, Razonable RR, Ressner R, Chen A, Chow B, Escota G, Herc E, Johnson A, Maves RC, Nnedu O, Clauss H, Kulkarni P, Pottinger PS, Serpa JA, Bhowmick T, Bittner M, Wooten D, Casanas B, Shnekendorf R, Blumberg EA (2020). The struggling infectious diseases fellow: remediation challenges and opportunities. Open Forum Infect Dis.

[30] Aram N, Brazil V, Davin L, Greenslade J (2013). Intern underperformance is detected more frequently in emergency medicine rotations. Emerg Med Australas.

[31] Parsons AS, Dreicer JJ, Martindale JR, Young G, Warburton KM (2024). A targeted clinical reasoning remediation program for residents and fellows in need. J Grad Med Educ.

[32] Guerrasio J, Garrity MJ, Aagaard EM (2014). Learner deficits and academic outcomes of medical students, residents, fellows, and attending physicians referred to a remediation program, 2006-2012. Acad Med.

[33] Yao DC, Wright SM (2000). National survey of internal medicine residency program directors regarding problem residents. JAMA.

[34] Bhatti NI, Ahmed A, Stewart MG, Miller RH, Choi SS (2016). Remediation of problematic residents: a national survey. Laryngoscope.

[35] Warburton KM, Goren E, Dine CJ (2017). Comprehensive assessment of struggling learners referred to a graduate medical education remediation program. J Grad Med Educ.

[36] Naude JM, Burch VC (2018). Checklist of cognitive contributions to diagnostic errors: a tool for clinician-educators. Afr J Health Prof Educ.

[37] Boyle SM, Martindale J, Parsons AS, Sozio SM, Hilburg R, Bahrainwala J, Chan L, Stern LD, Warburton KM (2024). Development and validation of a formative assessment tool for nephrology fellows’ clinical reasoning. Clin J Am Soc Nephrol.

[38] Audétat MC, Voirol C, Béland N, Fernandez N, Sanche G (2015). Remediation plans in family medicine residency. Can Fam Physician.

[39] Ellaway RH, Chou CL, Kalet AL (2018). Situating remediation: accommodating success and failure in medical education systems. Acad Med.

[40] Cheong CW, Quah EL, Chua KZ, Lim WQ, Toh RQ, Chiang CL, Ng CW, Lim EG, Teo YH, Kow CS, Vijayprasanth R, Liang ZJ, Tan YK, Tan JR, Chiam M, Lee AS, Ong YT, Chin AM, Wijaya L, Fong W, Mason S, Krishna LK (2022). Post graduate remediation programs in medicine: a scoping review. BMC Med Educ.

[41] Dudek NL, Marks MB, Regehr G (2005). Failure to fail: the perspectives of clinical supervisors. Acad Med.

[42] Patel R, Tarrant C, Bonas S, Yates J, Sandars J (2015). The struggling student: a thematic analysis from the self-regulated learning perspective. Med Educ.

[43] Castanelli DJ, Molloy E, Bearman M (2025). The stigma of underperformance in assessment and remediation. Adv Health Sci Educ Theory Pract.

[44] Chou CL, Kalet A, Costa MJ, Cleland J, Winston K (2019). Guidelines: the dos, don’ts and don’t knows of remediation in medical education. Perspect Med Educ.

[45] Ten Cate O, Regehr G (2019). The power of subjectivity in the assessment of medical trainees. Acad Med.

[46] Daniels L, Roosevelt G, Chadwick D, Tomberg S (2025). Measuring resilience, performance anxiety and burnout in emergency medicine residents. Educ Health.

[47] Schaller-Paule MA, Steinmetz H, Vollmer FS, Plesac M, Wicke F, Foerch C (2021). Lessons in clinical reasoning - pitfalls, myths, and pearls: the contribution of faulty data gathering and synthesis to diagnostic error. Diagnosis (Berl).

[48] Truong S, Rodrigue F, Culver D (2023). Collaborative narrative coaching in medicine: a case study with a resident physician and a practicing emergency physician. Can Med Educ J.

[49] Meunier MR, Theofiles MG, Gonzalez CA (2025). A structured approach to mitigating cognitive bias in educational assessment. J Grad Med Educ.

[50] Winston K (2015). Core concepts in remediation: lessons learned from a 6-year case study. Med Sci Educ.

