# Supplementary material for: Strategies for remediating clinical reasoning skill deficits in underperforming residents: a scoping review
Source: J Educ Eval Health Prof. 2026 Feb 5;23:3. doi: 10.3352/jeehp.2026.23.3 (PMC13039651; doi:10.3352/jeehp.2026.23.3)
Supplement: Supplementary file 2 — Supplement 2. Detailed search strategy. [file jeehp-23-03-suppl2.docx]

**Supplement 2.** Detailed search strategy

**MEDLINE – Search strategy**

Database: Ovid MEDLINE(R) and Epub Ahead of Print, In-Process, & Other Non-Indexed Citations

**P (Population)**: ONLY Medical Residents in Formal Training Programs

1. House Staff (MeSH)

2. Internship and Residency (MeSH)

3. Education, medical, graduate (MeSH)

4. resident*.mp.

5. intern*.mp.

6. fellow*.mp.

7. trainee*.mp.

8. "house officer*".mp.

9. "physician in training".mp.

10. "junior doctor*".mp.

11. 1 or 2 or 3 or 4 or 5 or 6 or 7 or 8 or 9 or 10

**I (Intervention)**: Remediation programs addressing clinical reasoning skills

**Part A: Remediation concepts**

12. exp Remedial Teaching (MeSH)

13. remediation.mp.

14. remedial.mp.

15. "academic support".mp.

16. "performance improvement".mp.

17. "academic difficulty".mp.

18. "resident in difficulty".mp.

19. "failing resident*".mp.

20. "struggling resident*".mp.

21. 12 or 13 or 14 or 15 or 16 or 17 or 18 or 19 or 20

**Part B: Clinical reasoning concepts**

22. Reasoning (MeSH)

23. "clinical reasoning".mp.

24. "critical thinking".mp.

25. "decision making".mp.

26. "clinical judgment".mp.

27. "problem solving".mp.

28. "diagnostic reasoning".mp.

29. 22 or 23 or 24 or 25 or 26 or 27 or 28

30. 21 and 29

**O (Outcome)**: Effectiveness of remediation programs in improving clinical reasoning skills

31. Program Evaluation (MeSH)

32. Competence (MeSH)

33. Clinical Competence (MeSH)

34. Educational Measurement (MeSH)

35. Performance (MeSH)

36. Academic Performance (MeSH)

37. effectiveness.mp.

38. efficacy.mp.

39. outcome*.mp.

40. impact.mp.

41. improve*.mp.

42. enhance*.mp.

43. assessment*.mp.

44. evaluation.mp.

45. 31 or 32 or 33 or 34 or 35 or 36 or 37 or 38 or 39 or 40 or 41 or 42 or 43 or 44

46. 11 and 30 and 45

47. Limit 46 to (English language and full text)

48. Limit 47 to (yr="2000 - 2024")

**Boolean operator – MEDLINE**

(House Staff/ OR Internship and Residency/ OR education, medical, graduate/ OR resident*.mp. OR intern*.mp. OR fellow*.mp. OR trainee*.mp. OR "house officer*".mp. OR "physician in training".mp. OR "junior doctor*".mp.) AND ((exp "Remedial Teaching"/ OR remediation.mp. OR remedial.mp. OR "academic support".mp. OR "performance improvement".mp. OR "academic difficulty".mp. OR "resident in difficulty".mp. OR "failing resident*".mp. OR "struggling resident*".mp.) AND (Reasoning/ OR "clinical reasoning".mp. OR "critical thinking".mp. OR "decision making".mp. OR "clinical judgment".mp. OR "problem solving".mp. OR "diagnostic reasoning".mp.)) AND (Program Evaluation/ OR Competence/ OR Clinical Competence/ OR Educational Measurement/ OR Performance/ OR Academic Performance/ OR effectiveness.mp. OR efficacy.mp. OR outcome*.mp. OR impact.mp. OR improve*.mp. OR enhance*.mp. OR assessment*.mp. OR evaluation.mp.)

Filter: English Language, Publication Year 2000 - 2024

**Boolean operator – PUBMED**

(("House Staff"[MeSH] OR "Internship and Residency"[MeSH] OR "Education, Medical, Graduate"[MeSH] OR resident*[tiab] OR intern*[tiab] OR fellow*[tiab] OR trainee*[tiab] OR "house officer*"[tiab] OR "physician in training"[tiab] OR "junior doctor*"[tiab])) AND (((Remedial Teaching/[MeSH] OR remediation[tiab] OR remedial[tiab] OR "academic support"[tiab] OR "performance improvement"[tiab] OR "academic difficulty"[tiab] OR "resident in difficulty"[tiab] OR "failing resident*"[tiab] OR "struggling resident*"[tiab])) AND (Reasoning[MeSH] OR "clinical reasoning"[tiab] OR "critical thinking"[tiab] OR "decision making"[tiab] OR "clinical judgment"[tiab] OR "problem solving"[tiab] OR "diagnostic reasoning"[tiab])) AND ("Program Evaluation"[MeSH] OR "Competence"[MeSH] OR "Clinical Competence"[MeSH] OR "Educational Measurement"[MeSH] OR "Performance"[MeSH] OR "Academic Performance"[MeSH] OR effectiveness[tiab] OR efficacy[tiab] OR outcome*[tiab] OR impact[tiab] OR improve*[tiab] OR enhance*[tiab] OR assessment*[tiab] OR evaluation[tiab])

Filter: English Language, Publication Year 2000 - 2024

**Boolean operator – SCOPUS**

(TITLE-ABS-KEY("House Staff" OR "Internship and Residency" OR "Education Medical Graduate" OR resident* OR intern* OR fellow* OR trainee* OR "house officer*" OR "physician in training" OR "junior doctor*")) AND (TITLE-ABS-KEY("Remedial Teaching" OR remediation OR remedial OR "academic support" OR "performance improvement" OR "academic difficulty" OR "resident in difficulty" OR "failing resident*" OR "struggling resident*") AND TITLE-ABS-KEY(Reasoning OR "clinical reasoning" OR "critical thinking" OR "decision making" OR "clinical judgment" OR "problem solving" OR "diagnostic reasoning")) AND (TITLE-ABS-KEY("Program Evaluation" OR Competence OR "Clinical Competence" OR "Educational Measurement" OR Performance OR "Academic Performance" OR effectiveness OR efficacy OR outcome* OR impact OR improve* OR enhance* OR assessment* OR evaluation))

Filter: English Language, Publication Year 2000 - 2024

**Boolean operator – SpringerLink**

("House Staff" OR "Internship and Residency" OR "Education medical graduate" OR resident* OR intern* OR fellow* OR trainee* OR "house officer*" OR "physician in training" OR "junior doctor*") AND (("Remedial Teaching" OR remediation* OR remedial* OR "academic support" OR "performance improvement" OR "academic difficulty" OR "resident in difficulty" OR "failing resident*" OR "struggling resident*") AND (Reasoning OR "clinical reasoning" OR "critical thinking" OR "decision making" OR "clinical judgment" OR "problem solving" OR "diagnostic reasoning")) AND ("Program Evaluation" OR Competence OR "Clinical Competence" OR "Educational Measurement" OR Performance OR "Academic Performance" OR effectiveness OR efficacy OR outcome* OR impact OR improve* OR enhance* OR assessment* OR evaluation)

Filter: English Language, Publication Year 2000 - 2024

**Boolean operator – ProQuest**

((ti("House Staff" OR "Internship and Residency" OR "Education medical graduate" OR resident* OR intern* OR fellow* OR trainee* OR "house officer*" OR "physician in training" OR "junior doctor*") OR ab("House Staff" OR "Internship and Residency" OR "Education medical graduate" OR resident* OR intern* OR fellow* OR trainee* OR "house officer*" OR "physician in training" OR "junior doctor*"))) AND ((ti("Remedial Teaching" OR remediation OR remedial OR "academic support" OR "performance improvement" OR "academic difficulty" OR "resident in difficulty" OR "failing resident*" OR "struggling resident*") OR ab("Remedial Teaching" OR remediation OR remedial OR "academic support" OR "performance improvement" OR "academic difficulty" OR "resident in difficulty" OR "failing resident*" OR "struggling resident*")) AND (ti(Reasoning OR "clinical reasoning" OR "critical thinking" OR "decision making" OR "clinical judgment" OR "problem solving" OR "diagnostic reasoning") OR ab(Reasoning OR "clinical reasoning" OR "critical thinking" OR "decision making" OR "clinical judgment" OR "problem solving" OR "diagnostic reasoning"))) AND ((ti("Program Evaluation" OR Competence OR "Clinical Competence" OR "Educational Measurement" OR Performance OR "Academic Performance" OR effectiveness OR efficacy OR outcome* OR impact OR improve* OR enhance* OR assessment* OR evaluation) OR ab("Program Evaluation" OR Competence OR "Clinical Competence" OR "Educational Measurement" OR Performance OR "Academic Performance" OR effectiveness OR efficacy OR outcome* OR impact OR improve* OR enhance* OR assessment* OR evaluation)))

Filter: English Language, Publication Year 2000 - 2024

**Boolean operator – EBSCOhost**

(MH "House Staff" OR MH "Internship and Residency" OR MH "Education, Medical, Graduate" OR resident* OR intern* OR fellow* OR trainee* OR "house officer*" OR "physician in training" OR "junior doctor*") AND ((MH "Remedial Teaching+" OR remediation OR remedial OR "academic support" OR "performance improvement" OR "academic difficulty" OR "resident in difficulty" OR "failing resident*" OR "struggling resident*") AND (MH "Reasoning" OR "clinical reasoning" OR "critical thinking" OR "decision making" OR "clinical judgment" OR "problem solving" OR "diagnostic reasoning")) AND (MH "Program Evaluation" OR MH "Competence" OR MH "Clinical Competence" OR MH "Educational Measurement" OR MH "Performance" OR MH "Academic Performance" OR effectiveness OR efficacy OR outcome* OR impact OR improve* OR enhance* OR assessment* OR evaluation)

Filter: English Language, Publication Year 2000 - 2024

**Boolean operator – Web of Science**

TS=("House Staff" OR "Internship and Residency" OR "Education Medical Graduate" OR resident* OR intern* OR fellow* OR trainee* OR "house officer*" OR "physician in training" OR "junior doctor*") AND (TS=("Remedial Teaching" OR remediation OR remedial OR "academic support" OR "performance improvement" OR "academic difficulty" OR "resident in difficulty" OR "failing resident*" OR "struggling resident*") AND TS=(Reasoning OR "clinical reasoning" OR "critical thinking" OR "decision making" OR "clinical judgment" OR "problem solving" OR "diagnostic reasoning")) AND TS=("Program Evaluation" OR Competence OR "Clinical Competence" OR "Educational Measurement" OR Performance OR "Academic Performance" OR effectiveness OR efficacy OR outcome* OR impact OR improve* OR enhance* OR assessment* OR evaluation)

Filter: English Language, Publication Year 2000 – 2024
