# Supplementary material for: Strategies for remediating clinical reasoning skill deficits in underperforming residents: a scoping review
Source: J Educ Eval Health Prof. 2026 Feb 5;23:3. doi: 10.3352/jeehp.2026.23.3 (PMC13039651; doi:10.3352/jeehp.2026.23.3)
Supplement: Supplementary file 3 — Supplement 3. Data extraction form. [file jeehp-23-03-suppl3.docx]

**Supplement 3.** Data extraction form

| Reviewer’s initial: |  |
| --- | --- |
| Review date: |  |
| Article title: |  |
| Last name of first author: |  |
| Location of author(s): |  |
| Type of article: | Mixed methods research  Meta-analyses  Systematic reviews  Randomised controlled trials  Cohort studies  Case-control studies  Cross-sectional studies  Descriptive/opinion papers (exclude)  Others, please specify……. |
| Inclusion criteria screening (only proceed if all three of the criteria are checked) | MUST discuss about resident/physician in training  MUST discuss remediation strategy  MUST discuss clinical reasoning |

| Answer the following questions based on the research article provided. Write N/A if not applicable. |
| --- |
| 1) What tools or criteria are used to identify residents with clinical reasoning skill deficits? |
| Answer: |
|  |
| 2) What remediation strategies have been implemented to address these deficits? |
| Answer: |
|  |
| 3) How effective are these remediation strategies? (Mention both qualitative or quantitative data provided in the study) |
| Answer: |
|  |
| 4) What factors facilitate or hinder the successful remediation of clinical reasoning skills in residents? |
| Answer: |
|  |
| 5) Briefly state the purpose of this article |
| Answer: |
|  |
| 6) Provide any additional observations or comments related to remediation of clinical reasoning deficits in residency training. |
| Answer: |
|  |
